# Environmental responsibility in the Israeli health system in the era of climate change: a required paradigm shift

**DOI:** 10.1186/s13584-025-00684-6

**Published:** 2025-05-01

**Authors:** Raanan Raz, Maya Negev, Michael Hauzer, Eliaz Miller, Ora Paltiel, Meidad Kissinger

**Affiliations:** 1 Braun School of Public Health, The Hebrew University of Jerusalem– Hadassah, Jerusalem, Israel; 2 School of Public Health, University of Haifa, Haifa, Israel; 3 Research and Innovation Unit, Clalit Health Services, Haifa district, Haifa, Israel; 4 Hospital Division, General Management, Clalit Health Services, Tel Aviv, Israel; 5 Department of Environmental, Geoinformatics and Urban Planning Sciences, Ben- Gurion University, Be’er Sheva, Israel

**Keywords:** Climate change, Greenhouse gases, Sustainability

## Abstract

**Background:**

Environmental management in the Israeli health system is driven primarily by safety regulations. Such regulations aim to reduce hazardous exposures to employees, patients, and visitors, as well as some specific aspects of broader environmental toxicity to humans and nature. Most environmental precautions in the system target traditional exposures and do not specifically consider the health system’s own impact on climate change. This article aims to justify incorporating climate change mitigation actions into short- and long-term plans in Israeli health organizations and present a schematic strategic roadmap to do so.

**Main body:**

Climate change poses many threats to global health, including risks from severe weather events, changes in vector-borne diseases, increased hazardous air pollutants, food and water shortages, and adverse effects on reproductive health. The most effective effort in climate change mitigation is reducing greenhouse gas emissions to the atmosphere. Ignoring the health sector’s emissions contradicts the ancient medical principle: first, do no harm (*primum non-nocere*). Furthermore, many climate mitigation methods introduce additional health co-benefits. Special attention and medical considerations are needed to safely reduce emissions from the health sector. This article reviews healthcare’s most common emission sources, including energy consumption, transportation, food, waste, supplies, and the supply chain. An organizational carbon management strategy should include recognizing the problem and committing to action, estimating the organizational carbon footprint, developing and prioritizing alternative interventions, and developing a carbon management plan with measurable short- and intermediate-term goals.

**Conclusion:**

Climate mitigation in the health sector is encompassed by the moral obligation of the Israeli healthcare system to do no harm. Performance measures to support GHG emission reductions should be adopted into the existing, successful Israeli programs of quality measures in medicine, both in the community and hospitals. In addition, Israel academic institutions for health and medical education should incorporate sustainable health into their curricula for students of health professions and as part of continuous medical education. Such policy actions will contribute to a healthy health system that supports climate change mitigation while providing health co-benefits to the Israeli population.

## Background

Many national health systems worldwide have recognized the critical link between environmental hazards and human health in recent years [1, 2]. As humanity has advanced and improved various aspects of quality of life, the resulting pressure on natural systems has intensified. This has led to the deterioration of ecosystems and accelerated climate change, posing significant risks to human health and threatening the sustainability of current and future generations. As major public institutions serving all segments of society, health systems also consume environmental resources and generate waste. Thus, they have an obligation to take an active role in promoting environmental health.

The Israeli health system is stretched to its limits and beyond, as demonstrated by the ratio of acute care beds to population size in hospitals, which is among the lowest in the OECD, with a high occupancy rate [3]. 

The system frequently encounters multiple emergencies, such as the October 7th attack and the subsequent war, which heighten operational stress and demand considerable managerial focus. Given the challenges of delivering health services daily, it is unsurprising that Israeli healthcare leadership has less attention to long-term considerations. This is particularly evident regarding the system’s long-term environmental impacts, often viewed as outside the primary obligations of healthcare providers. Conversely, the Israeli healthcare system is highly regulated, featuring a relatively small number of operators and extensive use of incentives and metrics programs, thus enabling long-term planning.

Currently, environmental management within the Israeli health system is primarily driven by safety regulations. These regulations aim to reduce hazardous exposures for employees, patients, and visitors, as well as address some specific aspects of broader environmental toxicity affecting humans and nature due to air, water, and soil pollution. In other words, the majority of environmental precautions in the system focus on traditional exposures and do not specifically consider the health system’s impact on climate change. This article aims to encourage the inclusion of climate change mitigation actions in both short- and long-term plans for Israeli health organizations and to present a schematic strategic roadmap.

## Main text

### Carbon emissions from health organizations: why should we care?

Climate change poses many threats to global health. For example, the frequency of severe weather events such as heatwaves, droughts, and floods is expected to increase, causing mortality and morbidity throughout the globe [4]. Extreme heat contributes to cardiovascular, cerebrovascular, and respiratory mortality and morbidity and is also associated with external causes of death, including injuries and violence. Climate change especially exacerbates the health vulnerability of sensitive populations, including older adults, people suffering from chronic diseases, people with low socioeconomic status, immigrants, and people who work outdoors, further expanding existing health disparities [4, 5]. 

Climate change may also affect public health less directly, influencing vectors of infectious diseases, women’s and children’s health, the spread of allergens, hazardous air pollutants such as fine particulate matter and ozone, food and water shortages, and other fundamental risk factors [4]. Accordingly, fighting against climate change is considered by public health experts to be the most critical global health intervention and medical practitioners are being called upon to take leadership in this public health emergency [6, 7]. 

There are two strategies for combating climate change: adaptation and mitigation. Adaptation in health systems aims to prepare for the already inevitable short- and intermediate-term changes in climate and their health implications. Health systems worldwide are responsible for adapting to the expected changes in morbidity in their region [8]. Mitigation, on the other hand, aims to slow and eventually stop climate change by gradually weakening the factors driving it. Since climate change is a progressive process, adaptation must be accompanied by mitigation, creating a stable climate to adapt to without further global warming.

The most effective effort in climate change mitigation is reducing emissions of greenhouse gases (GHGs) into the atmosphere [9]. GHGs are emitted from almost every human activity, including generating energy by burning fossil fuels, agriculture, industrial operations, transportation, mining of natural resources, waste, water consumption, and others [10]. Various techniques have been developed to quantify the amount of GHG emissions (a.k.a. “carbon footprint”) from a specific activity, service, or product lifecycle. Quantifying the direct and indirect emissions related to various operations of any organization can estimate the overall related emissions, and the share of specific components can be highlighted. This may lead to developing alternative mitigation strategies and detailed comparisons of their implications across various dimensions.

For global climate mitigation to succeed, all economic sectors must be included. Worldwide and increasingly also in Israel, various public, industrial, and commercial institutions are now accounting for their carbon footprint and advancing measures for climate mitigation. Unlike the industry, transportation, and energy sectors, healthcare is not widely perceived as a major GHG emitter; nevertheless, it is estimated to produce 4.4% of global GHG emissions [11]. Ignoring the sector’s emissions contradicts one ancient but still relevant medical principle: first, do no harm (*primum non-nocere*). Climate health action means generalizing this principle beyond the individual level - where it is practiced routinely - to the global health level, similar to other major global health challenges such as the fight against antibiotic resistance. Furthermore, given the serious ramifications of climate change on public health, the health system should show leadership in minimizing its contribution to this crisis. Precedents for this include health care leadership in smoking policy [12] or safe travel of newborns home from the hospital [13]. 

Many climate mitigation methods introduce additional health co-benefits beyond preventing diseases directly caused by climate change. For example, by switching from fossil fuels to renewable energy sources, one also avoids producing and emitting hazardous air pollutants such as particulate matter, nitrous dioxide, and carbon monoxide. These common pollutants are responsible for millions of deaths annually [14], adding to the risk of prevalent diseases such as asthma, lung cancer, chronic obstructive pulmonary disease, chronic heart disease, and many others [14]. Similarly, by promoting active travel, one increases routine physical activity– a fundamental component of preventive medicine. Furthermore, GHG emissions can be substantially reduced by converting red meat consumption to plant-based protein sources such as legumes– another shift contributing to cardiovascular and general health [15]. 

Accordingly, over 50 countries committed themselves in 2021 to decarbonizing their healthcare systems, and tens of others joined later [2]. England’s National Health System had already reduced its emissions by 62% as of 2019 (compared with the 1990 baseline). It also published a plan to become net-zero by 2040 for the care it provides and by 2045 across its entire emission scope [16]. In addition, more than 1,900 health organizations from more than 80 countries worldwide joined the Global Green Healthy Hospitals network, committing to reducing emissions and collaborating on this shared goal [17]. Finally, the Joint Commission International (JCI), a high-influence standards organization worldwide, has published its first sustainable healthcare certification [18]. This is especially relevant for Israeli hospitals since almost all maintain the JCI accreditation or are working toward this goal.

Despite its national pledges under the Paris Agreement and its commitment to achieving net-zero emissions in the energy sector by 2050, Israel lacks regulations, initiatives, or published plans aimed at reducing greenhouse gas (GHG) emissions from its health sector. Based on our involvement in climate and health committees and initiatives in Israel, we recognize that several hospitals have declared sustainability initiatives that will also contribute to GHG reduction. Currently, efforts primarily focus on energy efficiency and recycling. However, a significant information gap remains. To the best of our knowledge, no data is available regarding the actual impact of these initiatives on GHG emissions. This further highlights the urgent need to initiate climate change mitigation efforts within the Israeli health system.

### Sources of carbon emissions in health organizations

Healthcare emissions include common sources such as energy consumption, transportation, domestic waste, and procurement, as well as sector-specific sources such as anesthetic gases, metered-dose inhaler propellants, and biohazardous waste management. Special attention and medical considerations are needed to safely reduce emissions from these sector-specific sources. On the other hand, many climate mitigation actions in the sector, such as switching from single-use to multiple-use medical items, have been proven safe and economical [19]. Many sustainability initiatives can enhance patient experience and outcomes. For example, improving energy efficiency can lead to better temperature control and air quality in patient rooms, while reducing food waste can allow more resources to be directed toward higher-quality, nutritious meals [20]. When trade-offs exist, they must be analyzed separately for each activity by modeling and comparing the medical, sustainability, and budgetary implications among several mitigation interventions. The following sections briefly overview several of healthcare’s most common emission sources.

### Energy

Energy consumption is one of the primary sources of GHG emissions in healthcare, especially in hospitals. While renewable energy has a very low carbon footprint, energy generated from fossil fuels involves GHG emissions that can be assessed relatively easily, given the fuel used (i.e., coal, natural gas, liquid petroleum gas, and others). GHGs are emitted in every phase of the process, starting from the mining or pumping of the fuel through its processing, shipping, and, eventually– burning to convert its chemical energy to heat. The carbon intensity of an energy supplier, i.e., the amount of GHGs emitted per unit of supplied energy, can be easily calculated given the exact mix of energy sources it uses [21]. 

Energy consumption can be divided into electricity purchased from the grid and energy generated in-house. The carbon footprint of electricity consumed from the grid in Israel in 2024 is relatively high since Israel produces only ~ 10% of its electricity from renewable sources (mainly photovoltaic panels), compared with 30% of renewables globally [22]. Israeli hospitals usually generate additional energy in-house, primarily using natural gas or other fossil fuels to produce steam. Several hospitals in Israel have converted much of their in-house energy to natural gas in recent years. Unfortunately, natural gas, despite being promoted as “green” or “environmentally friendly,” is a fossil fuel, and its lifecycle involves the emission of methane– a highly potent GHG - during the production and transfer phases, as well as the emission of CO_2_ during the burning phase [23]. In addition, many hospitals use diesel generators as backup and routinely turn them on for checks.

There are three main strategies by which health organizations can reduce their energy-related carbon footprint. Firstly, it is easier for hospitals to control the energy mix they produce in-house. This allows them to increase the renewable share of their energy mix, mainly by rooftop solar panels. Additional space for solar panels may be generated by the roofing of parking lots and other open areas in the hospital for which shade is beneficial, such as walking paths. In addition, emergency generators can be converted to energy storage by lithium-ion batteries or other storage technologies, which have been substantially developed in recent years and continue to improve. Such a switch will prevent harmful air pollution from diesel generators and improve the efficiency of renewable energy sources in the hospital campus. A step in this direction was recently announced by Wolfson Hospital in the city of Holon, with an agreement to replace its diesel boilers with an electric process heat storage system [24]. 

Secondly, recent changes in the electricity market regulation in Israel have enabled consumers to choose their electricity vendor among various physical and virtual vendors. In many countries, vendors differ in the share of electricity they produce from renewable sources, and this difference may widen in the future as the national renewables share increases.

Thirdly, the energy-related carbon footprint can be decreased by reducing energy consumption. This can be achieved by a wealth of projects and strategies, including energy-efficient buildings, changes in technologies and setpoints of temperature control of spaces, more efficient use of high-consumption devices such as imaging machinery [25], proper management of common low-consumption devices such as personal computers [26], and checklists for turning off unused devices and lighting [27]. 

### Transportation

Most health services require a physical presence in clinics and hospitals. Such presence involves workers’ daily or shift commutes, as well as patient and visitor travel. Additional transportation involves business travel (including air travel), travel for scientific collaboration, emergency vehicles, and transferring various materials (such as biopsies and equipment) within the health system.

The carbon footprint of each travel depends on the type of vehicle, the energy source it uses, and the travel length. There are various strategies to reduce this footprint. Firstly, further developing telemedicine services and switching to online meetings when possible are the best environmental choices. Secondly, promoting the use of and access to public or organized transportation for employees, visitors, and patients may be a major step. Employees who do not occupy parking spots can be compensated, further encouraging use of alternative transport methods. Other examples promoting active travel include availability of rental bikes and the shading of walking paths. Supporting active travel through walking or cycling may reduce emissions while improving public health. Thirdly, when private cars must be used, there are two main ways to reduce their carbon footprint: encouraging employee carpools and supporting electric vehicles, for example, through accessible charging stations. Electric cars have a smaller footprint compared to fossil fuel vehicles, even with the current Israeli electric grid, since they use energy much more efficiently, and this difference is expected to increase in the future. Fourthly, patient travel can sometimes be reduced by combining several procedures into one visit. Lastly, the in-house healthcare vehicles fleet can be electrified similarly to efforts in other countries [28], and its drivers can be educated to avoid idling, reducing harmful air pollution as well.

### Food consumption

Food systems account for 26–34% of global GHG emissions, with emissions occurring throughout the food life cycle, from agriculture through processing and supply to consumption and waste [29, 30]. This substantial share of emissions arises from several processes. Methane emissions from livestock digestion and manure are associated with consuming products produced by ruminants, such as dairy and red meat. Nitrous oxide (N_2_O)—another potent GHG—is released as a by-product of fertilizer application (as well as from its use as an anesthetic gas). Additional emissions stem from converting land to agricultural use, mainly through deforestation in tropical regions. The transportation of farming and food products contributes further to emissions, and finally, the decomposition of food waste represents the last phase of emissions in the food life cycle [31]. Recent studies have begun analyzing and estimating the share of food consumption-related GHG emissions within hospital operations, indicating that 17–30% of total emissions in the studied hospitals originate from food consumption [32–34]. 

There are many ways to reduce GHG emissions from healthcare-served food. The main approaches on this front are changes in the menu for patients and employees, use of local food, reduction of food waste, and proper management of organic and food packaging waste. The carbon footprint of the food supply largely depends on the amount of animal-based products purchased, with red meat having one of the most extensive footprint per weight and nutritional value [35–37]. A transition to more sustainable food has important health co-benefits, as the most unsustainable products are also not recommended as part of a healthy diet [38]. For example, a sustainable and healthy intervention can include gradually replacing red meat with legume-based dishes.

Naturally, the shipment of food supplies involves transport-related emissions determined by the shipment method and distance. In addition, approximately 30% of the food worldwide is unused and eventually wasted [36], suggesting that there is probably room for more efficient procurement and management of food also in Israeli hospitals. Finally, in most Israeli hospitals, organic waste is not segregated and eventually finds itself in landfills with all other non-hazardous waste, where it is reduced in anaerobic conditions, emitting additional methane. Instead, organic waste can be segregated and treated by composting or energy-from-waste (EfW) systems that can provide energy for the hospital, reducing the associated GHG emissions [39, 40]. While we are unaware of a specific estimate for food waste in Israeli hospitals, the proportion of food wasted in the entire country is estimated at 38%, accounting for 1.4% of Israel’s gross domestic product [41]. Other relevant, updated Israeli data and materials about sustainable nutrition in Hebrew are continuously published by the Israeli Forum for Sustainable Nutrition [42]. 

### Solid waste management

The regular operation of a health center produces infectious, pharmaceutical, cytotoxic, chemical, and radioactive waste, as well as non-hazardous solid waste such as organic matter and domestic waste, including recyclables (paper, glass, packaging, and electronics). Hazardous waste streams require proper segregation and treatment to prevent unacceptable risks to employees, patients, and the environment. Non-hazardous waste also requires segregation and treatment to reduce its environmental impact, including GHG emissions.

Plastic, despite being perceived as a non-hazardous, inert material, commonly contains hazardous additives such as bisphenol-A and phthalates. In addition, plastic decomposes to micro- and nano-plastic, with residuals in air, water, food, and various human tissues [43–45]. A recent study demonstrated a clear association between microplastic particles in carotid plaque specimens and a higher incidence of cardiovascular events in patients undergoing carotid endarterectomy for asymptomatic carotid artery disease [46]. While plastic is an essential material in many products used in healthcare, Israeli hospitals and clinics also buy single-use utensils and other catering-related items that can be replaced by their multiple-use versions.

Rizan et al. analyzed the carbon footprint of eleven different healthcare waste streams from a hospital in the UK, demonstrating the effect of the type and the location of the treatment on GHG emissions [39]. For example, recycling reusable surgical instruments had a carbon footprint of 21 Kg CO_2_e (CO_2_ equivalent) per tonne of waste, while GHG emissions from high-temperature incineration were more than 50-fold higher (1,074 Kg CO_2_e/tonne). Alternative treatment methods, such as low-temperature incineration involving capturing and usage of the energy emitted from the process (energy from waste, EfW) or autoclave decontamination before low-temperature incineration with EfW, produced about a quarter and about a half of the emissions relative to high-temperature incineration, respectively. The study also demonstrates that the processes emitting more GHGs were more expensive, aligning sustainable waste management with cost savings [39]. 

Waste treatment should match the risk from each hazardous waste stream. However, the study shows that it is environmentally and economically beneficial to carefully segregate healthcare waste into streams so that each item will be treated safely and have the lowest carbon footprint and expenses. For example, general non-infectious offensive waste (such as non-infectious gloves, aprons, and incontinence pads) does not pose a similar risk as infectious waste arising from a patient known or suspected to have a disease caused by a microorganism or associated toxins. The latter differs from the higher risk of infectious waste contaminated with chemicals or pharmaceuticals, such as medicated intravenous bags/lines, contaminated syringes, etc.

The Israeli health system can reduce its carbon footprint without compromising safety by improving the segregation of the various streams mentioned above and regulating the most proper treatment. In addition, it can reduce its GHG emissions by recycling non-hazardous plastics, glass, paper, and cardboard packages. Such recycling requires smooth cooperation with the local authority and the recycling corporations. Unfortunately, healthcare institutions, similar to other public sector service suppliers in Israel, do not always enjoy such cooperation, and much of the recyclable waste is not segregated and is eventually destined for landfill. This results from regulatory problems regarding the recycling process in the public sector in Israel that require governmental intervention [47]. 

### Supplies and supply chain

While energy and transportation are well-known sources of emissions, 62–82% of the healthcare carbon footprint is caused by indirect emissions from other activities [16, 48]. These emissions occur upstream or downstream of healthcare facilities, and the organization’s supply chain is responsible for most of them. Despite being the majority of emissions, it is much more difficult to quantify them accurately or control them due to their indirect nature. Food consumption, discussed above, is one source of these emissions; other large sources include pharmaceuticals, medical equipment, and medical supplies. Within the pharmaceuticals category, some agents - such as anesthetic gases [49] and meter-dose inhaler propellants [50] - are potent GHGs, and the production and shipment of all supplies involve GHG emissions. The trend toward single-use medical equipment items further magnifies the problem.

Interventions to reduce supply chain-related emissions are often specific to products or processes. They may include better inventory management, identification and reduction of wastage (as commonly done for personal protection equipment, such as gloves) [51], replacement of single-use instruments with multiple-use ones, avoidance of excessive packaging, safe reuse of “single-use” items, minimizing the discarding of unused supplies (often mandated by government regulations), reprocessing or refurbishing of medical equipment, and others. In this context, health organizations can benefit from adopting best practices in sustainability from sectors such as manufacturing, tourism, and technology. For example, the lean management principles widely used in manufacturing could be applied to reduce waste and improve efficiency in healthcare operations [52]. 

In addition, many health organizations from the UK [53], the US [54, 55], and Europe [56] develop sustainable procurement strategies to improve their environmental impact. Such strategies encourage more efficient operations that save space, energy, and water, reduce costs, improve environmental health, and support resilience by creating shorter supply chains, preventing severe disruptions during emergencies or other crises. Some examples include consideration of the total cost of ownership, demanding carbon emission reports and carbon reduction plans from suppliers, and gradually adding environmental criteria into procurement via tendering and other mechanisms, sending a sustainability signal to the market [57, 58]. 

### The path forward: a climate mitigation strategy process

The Israeli healthcare system is mainly public, with most hospitals and large clinics providing services to the public through universal mandatory health insurance. Pricing and billing for most services are determined by a complex set of laws, regulations, and agreements, leaving very little room for providers to make adjustments. Prices are typically based on a Diagnosis Related Group (DRG) system, not accounting for additional costs associated with operating health institutions, such as facilities management or process improvements. As a result, there is little incentive to adopt sustainable processes and technologies, particularly if these do not yield a quick return on investment.

On the other hand, recognizing this inherent flaw, the Ministry of Health employs an extensive array of incentive and metrics systems to encourage healthcare facilities to incorporate quality improvement processes, follow staffing recommendations, uphold various regulations, etc. Such incentives may include dedicated budgets, publicized metrics, rankings, or regulations for standards such as the JCI or ISO. To date, very few incentive programs aim to improve sustainability or reduce hospitals’ environmental impact.

**Fig. 1:**
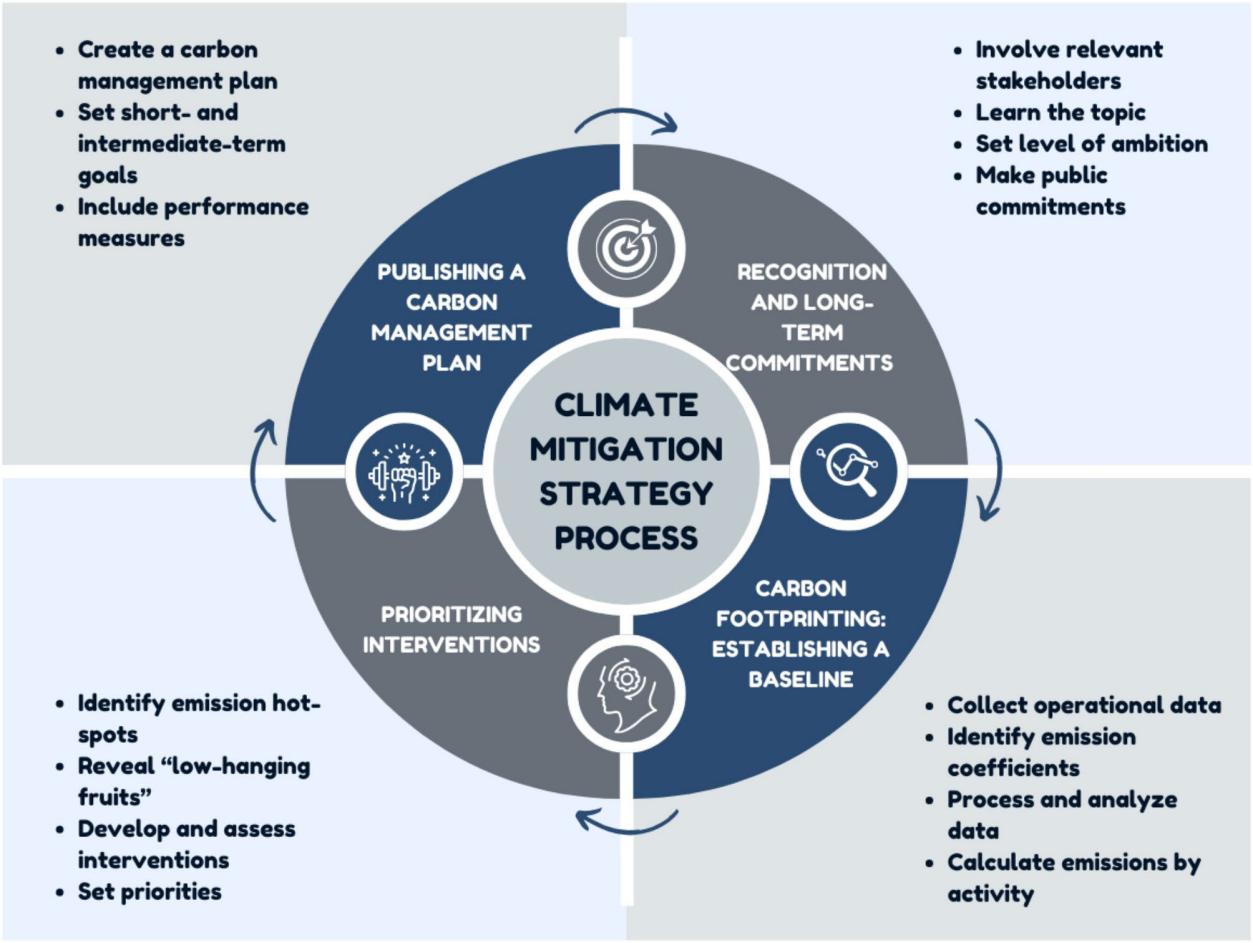
A simplified scheme for a climate mitigation strategy process

Designing climate interventions in the health sector is challenging due to institutional, economic, political, and informational factors [59]. The wealth of details about GHG emission reduction in healthcare facilities may be overwhelming. This section presents a simplified, general path for health organizations and managers who wish to be involved in climate mitigation but need to know where to start. This path includes a continuous organizational process of creating, monitoring, and updating a carbon management plan, with the ultimate goal of emission reductions based on short- and long-term measurable targets (Fig. 1). More detailed schemes and guidance can be found elsewhere [60]. 

The first phase on this path is recognizing the problem’s importance and relevance to the organization and committing to action. This step should involve key administrative, clinical, and infrastructure decision-makers and organizational stakeholders. It includes the joint learning of the topic and its relevance to various aspects of healthcare delivery, such as operating rooms, clinical wards, energy, waste management, procurement, and finance. It ends up with the setting and publishing of general but measurable long-term targets according to the level of ambition and commitment of the organization.

The second phase should estimate GHG emissions from all sources to establish a baseline since we cannot control what we do not measure. While there is no unified GHG protocol for healthcare, this phase will generally follow the emerging experience in various leading national and global healthcare emission reports [16, 61] and a growing number of academic publications [62, 63] with required technical adaptations to the Israeli settings that are beyond the scope of this article. It requires comprehensive data collection, processing, and analysis. Some of the data, such as energy and procurement data, should be extracted from the organizational information systems, but some of it may require on-site surveys (e.g., patient and staff transportation data) and cooperation of local municipalities and suppliers (e.g., solid waste amounts and composition).

The conversion of each activity into GHG emission units is based on the existing literature on health facilities worldwide [16, 34, 63–65], as well as on local and international databases (e.g., the eco-invent database; [66] GABI LCA software [67]), and often requires further research and development. Each activity is translated to emissions by multiplication in the best available emission factor. Such factors can be activity-based, such as that for fuel combustion, calculating emissions at the point of final activity. Alternatively, emission factors can be based on life-cycle analyses, accounting for emissions throughout various life-cycle stages of the product or activity, such as direct and indirect emissions related to hospital vehicles (‘Well to Wheel’). The analysis can also use some of the institution’s expenditure data on several activities and products to calculate related direct and indirect emissions by implementing an environmental input-output approach. The latter approach usually provides much less accurate estimates but is the only possibility for some activities. The final product of this phase is the organizational carbon footprint and its detailed distribution by specific activities.

The third phase comprises the identification of emission hotspots - major activities responsible for large portions of the carbon footprint - and “low-hanging fruits”– emissions that can easily, safely, and quickly be reduced. It involves the analysis of mitigation alternatives and their mitigation potential (in terms of emissions) while considering operational, patient and employee safety, patient care, and financial aspects, as well as factors that may limit or enhance the implementation. This information is then used to prioritize possible interventions to reduce the organizational carbon footprint.

While the specific interventions should be determined for each organization based on its data, approach, and priorities, common examples already implemented in various countries include investment in onsite renewable energy production and storage; improving medical waste segregation; proper prescription, collection, and disposal of metered-dose inhalers in the community; reducing the use of nitrous oxide in operation theatres; reducing consumption of disposable gloves while improving infection control; encouraging online consultation when physical presence is not needed; reducing the amount of red meat served in a hospital; and developing an extended sustainable procurement program.

The fourth and last phase includes developing and publishing a carbon management plan with measurable short- and intermediate-term goals. Such a plan incorporates all three first phases to implement the chosen interventions to fulfill the organizational commitment to reduce its carbon footprint from its baseline. Part of the plan is also the development of appropriate performance measures to be used as indicators for the progress of implementing each intervention. Such measures may include, for example, the average amount of nitrous oxide used per surgery, amounts of specific waste categories (with intentions to increase recyclables and reduce unneeded incineration or landfill), and measures related to reuse of medical equipment or decreased use of disposable items. Additional measures may be focused more directly on procurement, for example - the percentage of suppliers with carbon footprint data for their products/services or with published emission reduction plans.

### Barriers, facilitators, and plan re-evaluation

Consideration of barriers and facilitators is a crucial step toward the successful implementation of interventions. Such barriers and facilitators may be organizational, professional, individual, or cultural. They may include the local context, resources, perceived cost-benefit, organizational attention and constraints, staff awareness, agreement with the intervention, self-efficacy, and ability to modify the routine [68]. Relevant factors in this context may include inadequate education of staff members, building capacity and skills, initial investments for research and implementation of new interventions, and others [69]. Accordingly, a carbon management plan must be monitored and re-evaluated periodically, as should the organizational commitments, emission inventory, and interventions. This means that GHG emission reduction is not a linear process running from commitments to implementation; instead, it is a circular process in which obligations and plans continuously improve according to the updated organizational conditions, regulations, and norms.

### Conclusion and national implications

Health systems are in a constant process of long-term planning and construction of new buildings. Unfortunately, they have no clear incentives for long-term planning of supporting infrastructures to reduce their GHG emissions, such as advanced in-house waste treatment facilities, renewable energy projects, and low-emissions construction standards. In the coming years, two completely new major public medical centers will be built in Israel (in Be’er Sheva and the Haifa area), providing a rare opportunity for the Ministry of Health and all involved organizations to incorporate environmental principles already at the planning stage of these centers.

On a broader view, climate mitigation in the health sector is part of the moral obligation of the Israeli healthcare system to do no harm. This article aims to support the Israeli health system in preparing for the inevitable switch to a low-carbon economy, future accreditation standards and international regulations, and a more economically efficient use of resources. It is also relevant to national regulations related to energy, disposables, hospital waste, hospital nutrition, and other sub-fields. Some organizational performance measures to be developed to support GHG emission reductions may be adopted later on into the existing, successful Israeli programs of quality measures in medicine, both in the community and in hospitals.

Israeli academic institutions also have a key role in the transition to a low-carbon health system. Firstly, health and medicine faculties should incorporate sustainable health into their curricula for all health professions students and as part of continuous medical education. Increasing awareness and knowledge about climate change and its health impacts among healthcare workers and the general public is crucial [70]. Secondly, as research institutions, Israeli colleges and universities can initiate and manage research to gradually reduce the information and knowledge gap about the decarbonization of the health sector. For example, such research can involve creating and maintaining a database of energy sources and initiatives in hospitals, sustainable procurement efforts, and procured food. This can be done in collaboration with university hospitals and the Ministry of Health as part of the national quality indicator programs. These actions will contribute to a healthy health system that mitigates climate change and provides health co-benefits to the Israeli population.

## Data Availability

Not applicable.

## Abbreviations

GHGs: Greenhouse gases
JCI: Joint Commission International
EfW: Energy-from-waste
